# Tissue Plasminogen Activator Neurotoxicity is Neutralized by Recombinant ADAMTS 13

**DOI:** 10.1038/srep25971

**Published:** 2016-05-16

**Authors:** Mengchen Fan, Haochen Xu, Lixiang Wang, Haiyu Luo, Ximin Zhu, Ping Cai, Lixiang Wei, Lu Lu, Yongliang Cao, Rong Ye, Wenying Fan, Bing-Qiao Zhao

**Affiliations:** 1 State Key Laboratory of Medical Neurobiology, Collaborative Innovation Center for Brain Science, Shanghai Medical College and Institutes of Brain Science, Fudan University, Shanghai 200032, China; 2 Department of Microbiology and Parasitology, School of Basic Medical Sciences, Fudan University, Shanghai 200032, China

## Abstract

Tissue plasminogen activator (tPA) is an effective treatment for ischemic stroke, but its neurotoxicity is a significant problem. Here we tested the hypothesis that recombinant ADAMTS 13 (rADAMTS 13) would reduce tPA neurotoxicity in a mouse model of stroke. We show that treatment with rADAMTS 13 in combination with tPA significantly reduced infarct volume compared with mice treated with tPA alone 48 hours after stroke. The combination treatment significantly improved neurological deficits compared with mice treated with tPA or vehicle alone. These neuroprotective effects were associated with significant reductions in fibrin deposits in ischemic vessels and less severe cell death in ischemic brain. The effect of rADAMTS13 on tPA neurotoxicity was mimicked by the N-methyl-D-aspartate (NMDA) receptor antagonist M-801, and was abolished by injection of NMDA. Moreover, rADAMTS 13 prevents the neurotoxicity effect of tPA, by blocking its interaction with the NMDA receptor NR2B and the attendant phosphorylation of NR2B and activation of ERK1/2. Finally, the NR2B-specific NMDA receptor antagonist ifenprodil abolished tPA neurotoxicity and rADAMTS 13 treatment had no further beneficial effect. Our data suggest that the combination of rADAMTS 13 and tPA may provide a novel treatment of ischemic stroke by diminishing the neurotoxic effects of exogenous tPA.

Recent clinical trials provide evidence that endovascular intervention combined with medical management, including tissue plasminogen activator improves the outcomes of appropriately selected patients with acute ischemic stroke^1^,^2^. Tissue plasminogen activator (tPA) is an established treatment in acute ischemic stroke^3^,^4^. However, accumulating data indicate that, besides its beneficial thrombolytic role, tPA also has deleterious effects in the ischemic brain that can compromise the overall benefit from thrombolysis during stroke. Wang *et al.* first demonstrated that tPA-deficient mice showed smaller brain lesions after cerebral ischemia^5^. Subsequently, it was shown that administration of tPA resulted in a significantly increase in the volume of ischemic lesion after occlusion of the middle cerebral artery (MCA)^6^. Furthermore, studies showed that inhibition of tPA with PAI-1 or neuroserpin protected neurons against ischemic brain damage^7^,^8^. In addition, toxic effects of tPA has also been described during excitotoxin-induced neuronal cell death^9^. Although approaches aimed at diminishing the harmful effects of tPA may improve its thrombolytic efficacy, mechanisms leading to the neurtoxicity of tPA are not fully understood.

ADAMTS 13 (a disintegrin and metalloprotease with thrombospondin type I motif, member 13) is a protease that cleaves multimeric VWF (von Willebrand factor) into smaller units^10^, thereby preventing development of thrombotic thrombocytopenic purpura, a life-threatening disease characterized by the formation of microvascular thrombi^11^. Clinical studies revealed that low ADAMTS 13 plasma levels were associated with an increased risk of ischemic stroke and cardiovascular disease^12^,^13^. Likewise, animal studies have demonstrated that infusion of recombinant ADAMTS 13 (rADAMTS 13) resulted in a decrease in infarct volume after stroke and myocardial ischemia^14^,^15^, whereas genetic deficiency of ADAMTS 13 exacerbated ischemic brain injury^14^. We recently reported that injection of rADAMTS 13 significantly reduced tPA-mediated cerebral hemorrhage following ischemic stroke^16^. However, whether ADAMTS 13 plays a role in tPA neurotoxicity in the ischemic brain remains elusive.

In the present study, we hypothesize that combination of rADAMTS 13 with tPA would reduce the deleterious effects of delayed tPA administration. We demonstrated that treatment of rADAMTS 13 at 30 minutes and tPA at 4 hours after establishing reperfusion significantly reduced tPA-induced increase in infarct volume and reduced fibrin deposits. We further showed that rADAMTS 13 blocked the interaction between NR2B and exogenous tPA in the ischemic brain and thus reduced tPA-induced ischemic cell damage.

## Results

### Treatments with rADAMTS 13 alone and in combination with tPA reduced lesion volume and improved neurological deficits

**Figure 1:**
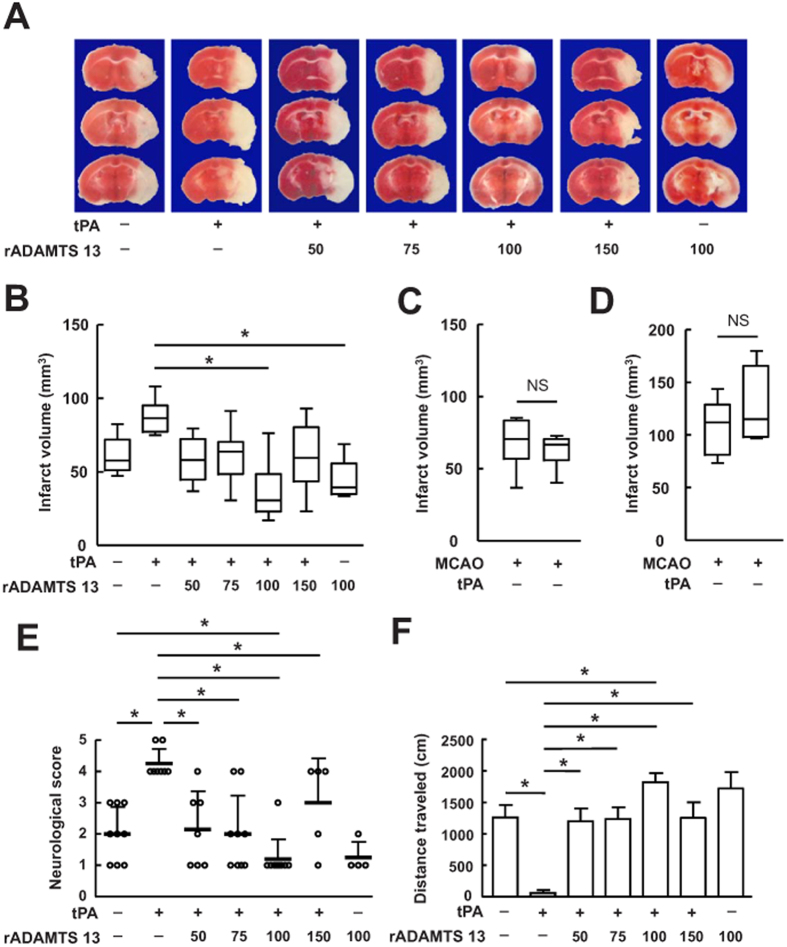
Treatments with rADAMTS 13 alone and in combination with tPA reduced ischemic lesion volume and improved behavioral outcome after MCA occlusion. (**A**) Photographs of TTC stained coronal brain section in representative mice treated with vehicle, tPA, increasing doses of rADAMTS 13 in combination with tPA, and rADAMTS 13 48 hours after MCA occlusion. (**B**) Quantitative analysis of infarct volume for each group. Values are mean ± SD (n = 8 per group). *P < 0.05. Combination treatment with 100 ng of rADAMTS 13 and tPA significantly reduced infarct volume compared with vehicle-treated and tPA-treated group. (**C**) Quantitative analysis of infarct volume in mice treated with vehicle or tPA at the onset of reperfusion. Values are mean ± SD (n = 6 per group). (**D**) Quantitative analysis of infarct volume in mice treated with vehicle or tPA in permanent ischemic model. Values are mean ± SD (n = 4 – 5 per group). (**E**,**F**) Neurological severity score (**E**) and locomotor activity (**F**) 48 hours after MCA occlusion in mice treated with vehicle, tPA, increasing doses of rADAMTS 13 in combination with tPA, and rADAMTS 13. Values are mean ± SD (n = 8 per group). *P < 0.05.

We firstly studied the effect of rADAMTS 13 on infarct volume after MCA occlusion in mice treated with tPA. tPA administrated intravenously at 4 hours after reperfusion moderately increased the infarct volume (Fig. 1A,B) and significantly worsened the neurological deficits (Fig. 1E,F) examined by the neurological scores and motor function 48 hours after stroke, consistent with earlier reports with tPA in this experimental model^17^.

Infusion of tPA at the onset of reperfusion did not affect infarct volume (Fig. 1C), as reports^18^. In the permanent ischemic model, infusion of tPA at 4 hours after MCA occlusion did not significantly increased infarct volume (Fig. 1D). Administration of rADAMTS 13 at 50, 75, 100, and 150 ng reduced infarct volume in tPA-treated mice, and saturates at a point (100 ng) where higher concentrations of rADAMTS 13 do not have any additional effect. Combination treatment with 100 ng of rADAMTS 13 and tPA significantly reduced infarct volume (by 57.93% of tPA alone; by 39.67% of control; (Fig. 1A,B) and improved neurological deficits (Fig. 1E,F) compared to that in tPA-treated or vehicle-treated mice. Monotherapy with rADAMTS 13 moderately reduced the infarct volume, as reported^14^.

### Recombinant ADAMTS 13 avoided fibrin deposits and reduced tPA-mediated ischemic cell death

**Figure 2:**
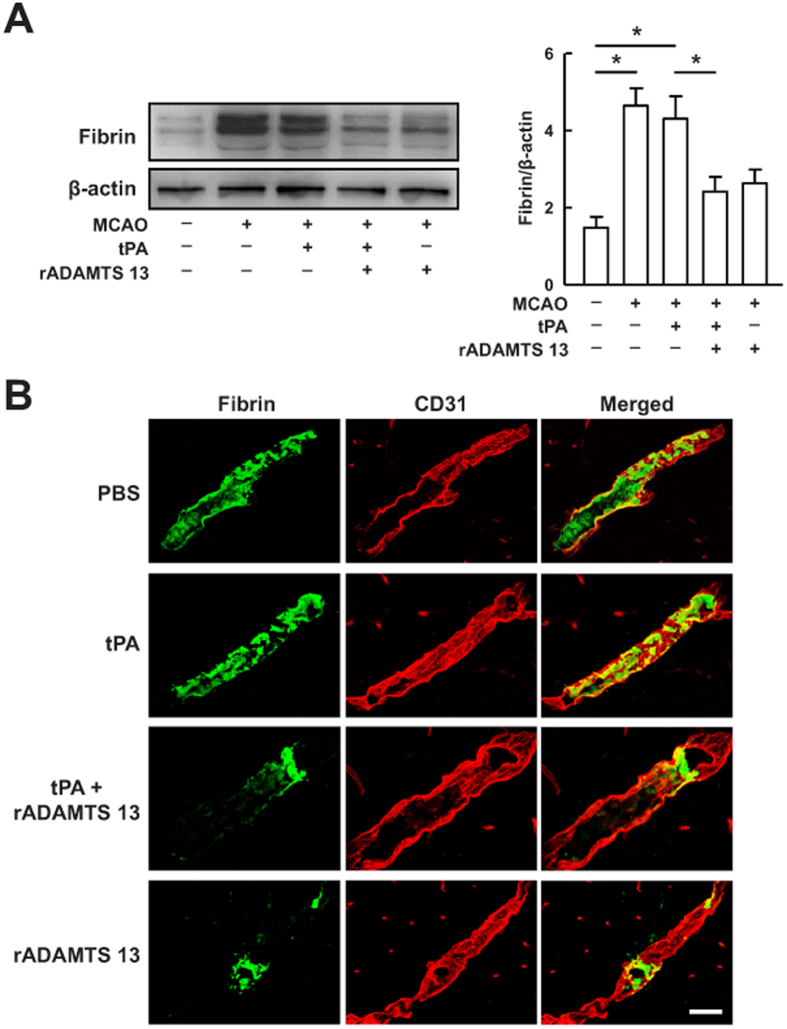
Treatment with rADAMTS 13 in combination with tPA reduced intracerebral thrombosis after MCA occlusion. (**A**) Representative immunoblots and quantitative analysis of fibrin in sham-operated mice, and mice treated with vehicle, tPA, tPA in combination with rADAMTS 13, and rADAMTS 13 24 hours after MCA occlusion. The gels have been run under the same experimental conditions. Values are mean ± SD (n = 5 per group). *P < 0.05. (**B**) Confocal microscopy images of fibrin(green)- and CD31-positive microvessels (red) in the ischemic hemispheres of mice treated with vehicle, tPA, rADAMTS 13 in combination with tPA, and rADAMTS 13 24 hours after MCA occlusion. Bar = 30 μm. Representative pictures from three independent experiments are shown.

To investigate whether reduced intracerebral fibrin deposits was responsible for the observed rADAMTS 13 effects on ischemic brain injury, we performed Western blot analysis with antibody that detects fibrin in the ischemic hemispheres 24 hours after MCA occlusion. As shown in Fig. 2A, cerebral ischemia resulted in a sustained 2.5-fold increase in the expression of fibrin. Intravenous injection of tPA 4 hours after reperfusion failed to reduce the expression of fibrin compared with PBS-treated mice. However, combination treatment with rADAMTS 13 and tPA significantly reduced the expression of fibrin in the ischemic hemisphere compared with the PBS treatment. Immunohistochemical analysis found that monotherapy with tPA had no effects on intravascular fibrin deposits, whereas rADAMTS 13 treatment alone eliminated fibrin deposits compared with PBS-treated mice (Fig. 2B). The combination treatment significantly reduced fibrin deposits compared with PBS-treated mice, consistent with the results of Western blot.

**Figure 3:**
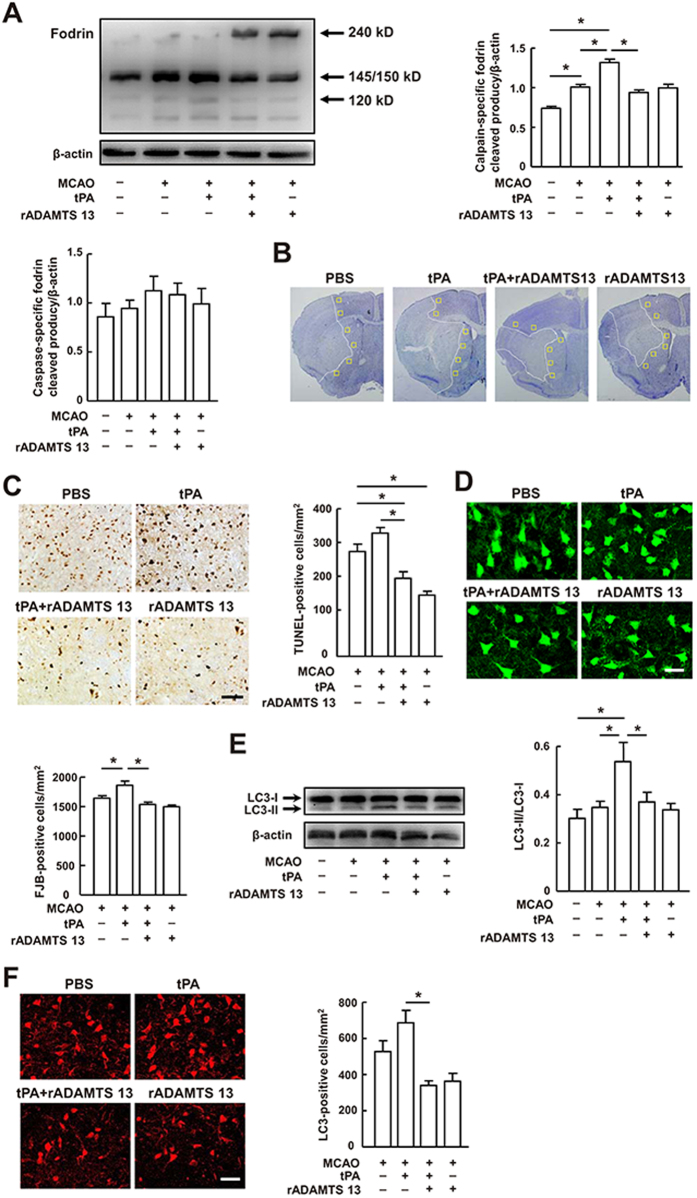
Recombinant ADAMTS 13 blocked tPA-induced apoptotic, necrotic, and autophagic cell death after MCA occlusion. (**A**) Representative immunoblots and quantitative analysis of the calpain- (145/150 kDa) and caspase-specific fodrin cleaved products (120 kDa) in sham-operated mice, and mice treated with vehicle, tPA, tPA in combination with rADAMTS 13, and rADAMTS 13 24 hours after MCA occlusion. The gels have been run under the same experimental conditions. (**B**) Six fields selected for immunohistochemical quantification of LC3, TUNEL, and FJB-labeled cells in the peri-infarct area in Nissl-stained coronal sections in all the groups. (**C**) Representative photomicrographs of TUNEL staining and quantification of TUNEL-positive cells in the ischemic brain of mice treated with vehicle, tPA, rADAMTS 13 in combination with tPA, and rADAMTS 13 24 hours after MCA occlusion. (**D**) Representative photomicrographs of Fluoro-Jade B (FJB) staining and quantification of FJB-positive cells in the ischemic brain of mice treated with vehicle, tPA, rADAMTS 13 in combination with tPA, and rADAMTS 13 24 hours after MCA occlusion. (**E**) Representative immunoblots of LC3 and quantification of the LC3-II/LC3-I ratio in sham-operated mice, and mice treated with vehicle, tPA, tPA in combination with rADAMTS 13, and rADAMTS 13 24 hours after MCA occlusion. The gels have been run under the same experimental conditions. (**F**) Representative photomicrographs of LC3 staining and quantification of LC3-positive cells in the ischemic brain of mice treated with vehicle, tPA, rADAMTS 13 in combination with tPA, and rADAMTS 13 24 hours after MCA occlusion. Bar = 30 μm. Values are mean ± SD (n = 5 per group). *P < 0.05.

We next studied whether the neuroprotective action of rADAMTS 13 is associated with reduced ischemic cell death. First, we evaluated the effects of rADAMTS 13 on apoptotic cell death in our stroke model. Western blots showed that cerebral ischemia elevated the levels of calpain-specific fodrin cleavage products compared with sham-operated group (Fig. 3A), as reported^19^. Treatment with tPA resulted in even greater increase in calpain-specific fodrin cleavage products compared with vehicle-treated mice. However, tPA treatment did not significantly increased the caspase-specific fodrin cleavage product (Fig. 3A). We also observed TUNEL-positive cells in the peri-infarct areas were moderately increased from 284.4 ± 45.81 in vehicle-treated mice to 334.4 ± 34.12 in tPA-treated mice (Fig. 3C). rADAMTS 13 diminished the calpain-specific fodrin cleavage products and TUNEL-positive cells in tPA-treated mice (Fig. 3A,C). rADAMTS 13 alone significantly reduced the number of TUNEL-positive cells, but did not affect the fodrin cleavage products. Then, we examined the capacity of rADAMTS 13 to regulate neuronal cell death. At 24 hours after stroke, brain sections were stained with FJB, a marker for degenerating neurons. Treatment with tPA alone significantly increased the number of FJB-positive neurons in the peri-infarct areas, but this effect was inhibited by rADAMTS 13 (Fig. 3D). We also analyzed autophagic cell death 24 hours after stroke, and found that the LC3-II/LC3-I ratio in mice treated with tPA was substantially reduced by >30% by rADAMTS 13, indicating that rADAMTS 13 markedly inhibits tPA-induced conversion from LC3-I to LC3-II (Fig. 3E). Immunocytochemistry using an anti-LC3 antibody confirmed that rADAMTS 13 reduced the number of LC3-positive cells in the peri-infarct areas compared to those in tPA-treated mice (Fig. 3F). Together, these results indicate that rADAMTS 13 improved ischemic cell injury induced by tPA administration after stroke.

### Recombinant ADAMTS 13 blocked tPA-induced ischemic injury via NR2B-containing NMDA receptor*-*dependent mechanism

**Figure 4:**
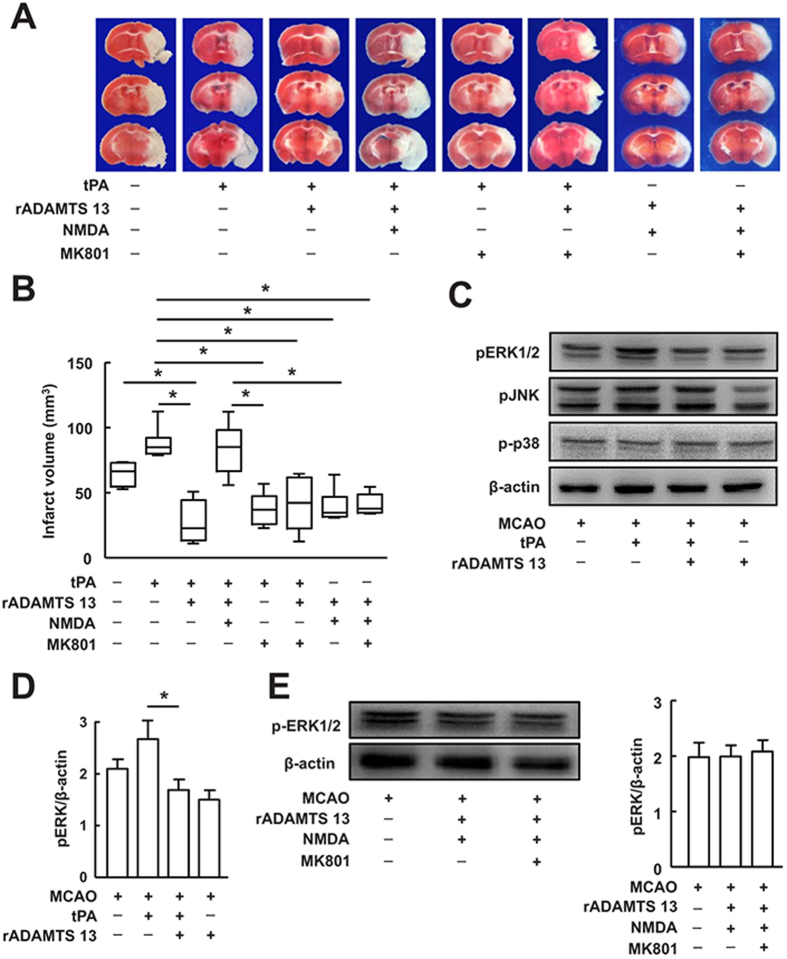
Recombinant ADAMTS 13 reduced tPA-mediated ischemic brain injury through NMDA receptors pathway. (**A**) Photographs of TTC stained coronal brain section in representative mice treated with vehicle, tPA, tPA + rADAMTS 13, tPA + rADAMTS 13 + NMDA, tPA + MK801, tPA + rADAMTS 13 + MK801, rADMTS13 + NMDA and rADMTS13 + NMDA + MK801 48 hours after MCA occlusion. (**B**) Quantitative analysis of infarct volume for each group. Values are mean ± SD (n = 6 – 8 per group). *P < 0.05. (**C**) Representative immunoblots of phosphorylated ERK1/2 (pERK), pJNK and p-p38 in mice treated with vehicle, tPA, tPA in combination with rADAMTS 13, and rADAMTS 13 24 hours after MCA occlusion. The gels have been run under the same experimental conditions. (**D**) Quantitative determinations of the levels of pERK for each group. Values are mean ± SD (n = 5 per group). *P < 0.05. (**E**) Representative immunoblots and quantification of pERK in mice treated with vehicle, rADMTS13 + NMDA and rADMTS13 + NMDA + MK801 24 hours after MCA occlusion. Values are mean ± SD (n = 5 per group).

Activation of NMDA receptor subtype is thought to play a crucial role in tPA-mediated excitoxic neuronal cell death^20^. To determine whether NMDA receptor is involved in the effect of rADAMTS 13 on tPA-induced neurotoxicity after stroke, we injected NMDA or MK801 in mice treated with tPA with or without rADAMTS 13. NMDA reversed the alteration of infarct volume in mice treated with tPA together with rADAMTS 13, whereas MK801, a non-competitive NMDA receptor antagonist, blocked tPA-induced increase in infarct volume (Fig. 4A,B). MK801 did not have any substantial effect on brain infarction in mice treated with tPA together with rADAMTS 13. The infarct volume was smaller in mice treated with rADMTS13 + NMDA and rADMTS13 + NMDA + MK801 than in mice treated with tPA + rADMTS13 + NMDA. There was no difference in infarct volume among mice treated with tPA + rADMTS13, mice treated with tPA + MK801, mice treated with tPA + rADMTS13 + MK801, mice treated with rADMTS13 + NMDA, or mice treated with rADMTS13 + NMDA + MK801. The levels of ERK1/2 phosphorylation were comparable among control group, mice treated with rADMTS13 + NMDA, or mice treated with rADMTS13 + NMDA + MK801. These findings suggest that rADAMTS 13 may downregulate tPA-induced ischemic brain injury through the NMDA receptor.

Phosphorylation of ERK1/2, has been considered a downstream step leading to NMDA receptor-mediated excitotoxicity^21^,^22^. We found that treatment of mice with rADAMTS 13 effectively reduced tPA-mediated ERK1/2 phosphorylation (Fig. 4C,D), lending further support to a potential role of NMDA receptor activation in rADAMTS 13-mediated inhibition of tPA-induced neurotoxicity. The levels of ERK1/2 phosphorylation were comparable among control group, mice treated with rADMTS13 + NMDA, or mice treated with rADMTS13 + NMDA + MK801 (Fig. 4E). In addition, rADAMTS 13 did not influence the levels of pJNK and p-p38 mitogen-activated protein kinases, both when administered alone and in combination with tPA (Fig. 4C).

**Figure 5:**
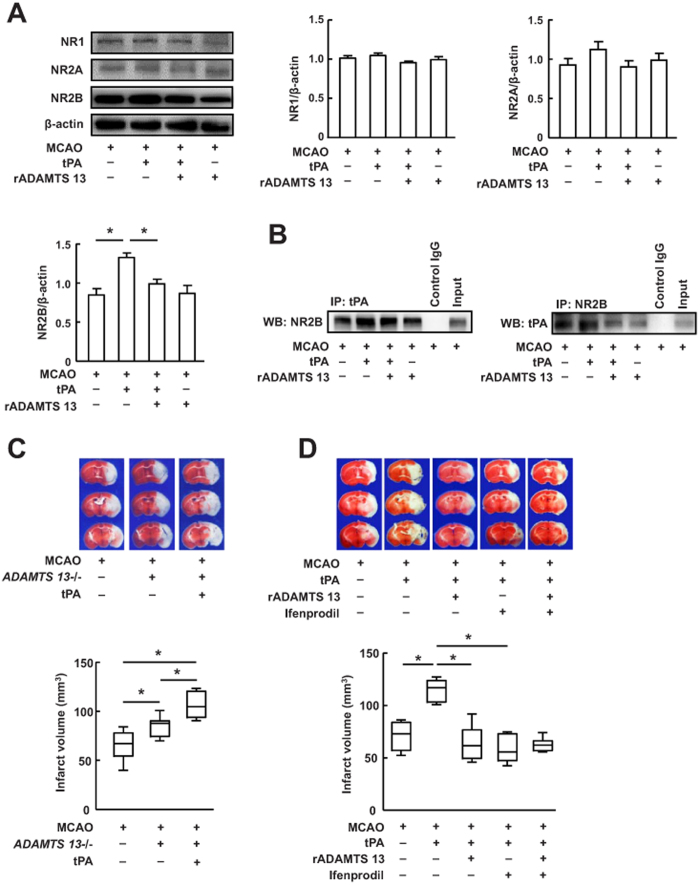
Recombinant ADAMTS 13 blocked tPA neurotoxicity by reducing exogenous tPA/NR2B interactions and the activation of NR2B. (**A**) Representative immunoblots and quantitative determinations of the levels of NR1, NR2A, and NR2B in mice treated with vehicle, tPA, tPA in combination with rADAMTS 13, and rADAMTS 13 24 hours after MCA occlusion. The gels have been run under the same experimental conditions. Values are mean ± SD (n = 5 per group). *P < 0.05. (**B**) Interaction between tPA and NR2B detected by immunoprecipitation analysis in ischemic brain of mice treated with vehicle, tPA, rADAMTS 13 in combination with tPA, and rADAMTS 13 24 hours after MCA occlusion. IP, immunoprecipitation; WB, Western blotting. Input, protein of the extracts without IP. The gels have been run under the same experimental conditions. Data are representative of three independent experiments. (**C**) Representative photographs of TTC stained coronal section and quantitative analysis of infarct volume in wild-type, *ADMTS13*^−/−^ mice and *ADMTS13*^−/−^ mice treated with tPA. Values are mean ± SD (n = 8 per group). *P < 0.05. (**D**) Representative photographs of TTC stained coronal section and quantitative analysis of infarct volume in mice treated with vehicle, tPA, tPA + rADAMTS 13, tPA + ifenprodil, and tPA + rADAMTS 13 + ifenprodil 48 hours after MCA occlusion. Values are mean ± SD (n = 8 per group). *P < 0.05.

To investigate which NMDA receptors are regulated by tPA, we measured the expression of NMDA receptor subunit NR1, NR2A and NR2B in the ischemic hemispheres of mice 24 hour after stroke by Western blot. tPA treatment substantially promoted NR2B expression, but not NR1 and NR2A (Fig. 5A), suggesting that NR2B is the predominant subunit during tPA neurotoxicity after stroke. tPA-induced expression of NR2B was significantly inhibited by rADAMTS 13 (Fig. 5A). To test whether tPA directly interacts with NR2B during stroke, we performed coimmunoprecipitation in extracts from the ischemic hemispheres of mice treated with or without tPA by using antibodies against NR2B and tPA. We found that NR2B coimmunoprecipitated with tPA in the vehicle-treated samples (Fig. 5B), as reported^23^. Mice treated with tPA showed a significantly higher interaction between NR2B and tPA than vehicle-treated samples. Although there was no detectable difference in NR2B/tPA interactions in mice treated with vehicle and in mice treated rADAMTS 13 alone, there was dramatically less of this interaction in mice treated tPA together with rADAMTS 13 than in mice treated with tPA alone. These results indicate a pivotal role for rADAMTS 13 in the interaction between exogenous tPA and NR2B. In order to demonstrate the interaction between ADMTS13 and tPA, we studied whether treatment with tPA affects infarct volume in *ADMTS13*^−/−^ mice. Our results showed that *ADMTS13*^−/−^ mice exhibited significantly increased infarct volume compared with wild-type mice (Fig. 5C), as reported^14^. Treatment with tPA resulted in even greater increase in the infarct volume compared with wild-type mice and vehicle-treated *ADMTS13*^−/−^ mice, indicating that ADMTS13 may interact with tPA during stroke. Finally, we showed that the NR2B subtype-specific antagonist ifenprodil, similar to rADAMTS 13, attenuated ischemic brain injury in tPA-treated mice (Fig. 5D). When both ifenprodil and rADAMTS 13 were combined, the tPA-induced increase of infarct volume was reduced to an extent that was similar to that seen with either treatment alone. These results suggest that the effect of rADAMTS 13 on tPA-induced ischemic brain injury is mediated via NR2B.

## Discussion

In this study, we showed that rADAMTS 13 combined with tPA not only blocked tPA-mediated neurotoxicity but also improved behavioral outcome after stroke in mice. Our data demonstrated that rADAMTS 13 protected from tPA-mediated neurotoxicity by diminishing the interaction between NR2B and exogenous tPA, the activation of NR2B, and downstream pERK1/2 signaling. We also showed that reduction of fibrin deposits in microvessel contributed to the benefits observed in the combination treatment.

tPA is used as thrombolytic therapy for ischemic stroke. Animal stroke studies indicate that cerebral ischemia results in extravasation of tPA^8^ into the brain tissue associated with neuronal death^5^. Furthermore, the neurovascular toxic effects of tPA have also been observed in stroke patients^24^. Our data indicate that delayed administration of tPA at 4 hours after reperfusion did not prevented fibrin deposits in ischemic vessels, but increased brain infarctions compared to controls. We further observed that tPA treatment resulted in more severe ischemic cell death in the peri-infarct areas. These results suggest that the toxic effect of tPA, such as stimulation of NMDA receptor or low-density lipoprotein receptor-related protein on ischemic cell, may have emerged and expanded infarctions in tPA-treated mice^25^,^26^. tPA may not exert its neurotoxic effects via its interaction with plasminogen, as mice deficiency of plasminogen showed exacerbated brain injury following stroke^27^. In contrast, We found that combination of rADAMTS 13 and tPA or monotherapy with rADAMTS 13 significantly reduced infarct volume and improved neurological function compared with mice treated with tPA alone, which was associated with significant reductions in fibrin deposits and less severe cell death in the ischemic brain. Further studies are warranted to investigate whether the reduced release of tPA by rADAMTS 13 in the ischemic brain may contribute to the neuroprotection of the combination treatment. Together, these data indicate that rADAMTS 13 neutralized the neurotoxicity of tPA and avoided fibrin deposits, which contributed to neuroprotection of the combination treatment after stroke.

Glutamate is the main excitatory neurotransmitter in the mammalian central nervous system^28^. Excessive release of glutamate is considered as a primary intracellular events that leads to neuronal toxicity and death in stroke^29^. NMDA receptors constitute the major subtype of glutamate receptors, and was associated with excitotoxin-mediated increase in ischemic neuronal death^30^,^31^. It has been shown that tPA potentiated NMDA-induced neuronal cell death^32^,^33^. Thus, tPA-mediated facilitation of excitotoxicity in the ischemic brain may involve direct or indirect interactions of tPA with NMDA receptor. Our study is consistent with this these principles, because tPA-induced ischemic brain injury was abrogated by MK801, a specific inhibitor of NMDA receptor. Our results also showed that treatment with NMDA reversed the effect of rADAMTS 13 on tPA-induced enhancement of infarct volume. These results suggest that the neuroprotection of the combined treatment of rADAMTS 13 with tPA was largely mediated by its ability to inhibit NMDA-mediated excitotoxic cell death. We further identified NMDA receptor NR2B subunit as a specific substrate of tPA within the ischemic brain. NR2A- and NR2B-containing NMDA receptor are considered as the main types of functional NMDA NR2 receptor in central nervous system neurons^34^. Blockade of NR2A enhanced neuronal death after global ischemia^35^, whereas NR2B-specific antagonist ifenprodil attenuated ischemic cell death^36^. Experimental evidence indicated that tPA interacted with the NR2B subunit of the NMDA receptor, and this NR2B-tPA complex played a significant role during ethanol withdrawal-induced seizures and acute stress^23^,^37^. Here we showed that delayed administration of tPA enhanced the NR2B-tPA interaction and activated NR2B-pERK1/2 signaling pathways. These events promoted tPA-mediated ischemic brain injury. Diminishing the interaction between NR2B and exogenous tPA by rADAMTS 13 inactivated NR2B and ERK1/2, and led to reduced excitoxic and ischemic injuries after late thrombolysis. These data suggest that that disturbing NR2B-tPA interaction would be able to decrease NMDA receptor NR2B toxicity mediated by exogenous tPA. Future studies are needed to determine how rADAMTS13 reduced the interaction between NR2B and tPA.

In summary, our data here showed that targeting the interaction of exogenous tPA with NMDA receptor NR2B by rADAMTS 13 counteracting the toxic effects of tPA on ischemic brain. Our previous results demonstrated that rADAMTS 13 could reduce tPA-associated cerebral hemorrhage after ischemic insult^16^. We suggest that treatment with tPA in combination with rADAMTS 13 may provide a new therapeutic strategy to avoid the neurotoxic effects of tPA and increase its safety for ischemic stroke.

## Methods

### Transient focal cerebral ischemia

All the described methods were carried out in accordance with the approved guidelines. All animal experiments were performed in accordance and approved by the Institutional Animal Care and Use Committee of Shanghai Medical College and Institutes of Brain Science, Fudan University. Adult male C57BL/6J mice (weighing 23 to 26 g; Shanghai SLAC Laboratory Animal Co. Ltd., China) were used. The ADAMTS 13-deficient mice used in this study were on a C57BL/6J background and were obtained from the Jackson Laboratory. A commonly studied filament model of experimental stroke was used in this study because previous studies have shown that administration of tPA increases neurotoxic effects in this model^17^,^38^. While embolic model of MCAO is more clinically relevant, a disadvantage of the model is that reproducibility is low^18^. The MCA was occluded as reported previously^16^,^39^. Briefly, Mice were anesthetized with 1–1.5% isoflurane in 30% oxygen. Rectal temperature was maintained at 37 ± 0.5 °C using a heating pad. A 7.0 siliconized monofilament was introduced into the right internal carotid artery through the external carotid artery to occlude the MCA. After occlusion for 45 minutes, the filament was withdrawn to restore blood flow. All mice were assessed with laser doppler flowmetry (Perimed, Stockholm, Sweden) to confirm successful occlusions and reperfusion. In preliminary experiments, we examined different duration of MCA occlusion. To reduce unacceptably high mortality rates, the 45-min transient MCA occlusion was used. Previous studies have shown that fibrin accumulated in the ischemic cerebral hemisphere after 45 minutes of MCA occlusion and 23 hours of reperfusion, suggesting that microvascular thrombi continue to accumulate even after recanalization of the MCA^40^. Thirty minutes after reperfusion, rADAMTS 13 (50, 75, 100, and 150 ng in 3 μl PBS; R&D systems, Minneapolis, MN, USA) or PBS was injected into the right lateral ventricle (0.2 mm posterior to bregma, 1.0 mm lateral to midline and 3.0 mm ventral to skull surface). Four hours after reperfusion, tPA (10 mg/kg, Actilyse, Boehringer Ingelheim, Mannheim, Germany) or PBS was administered as an intravenous bolus injection of 1 mg/kg followed by a 9 mg/kg infusion for 30 minutes with a syringe infusion pump (World Precision Instruments, Sarasota, FL, USA). NMDA (2 nM in 2 μl PBS, Sigma) or PBS was injected 30 minutes before MCA occlusion into the right lateral ventricle^41^. The relatively high dose of tPA was chosen on the basis of the approximately 10-fold difference in fibrin-specific activity between human and rodent system^42^, as well as previous thrombolytic doses used in studies of rt-PA in rodents^43^,^44^. However, this increased doses could be responsible for the neurotoxicity. MK-801 (0.2 mg/kg, Sigma-Aldrich, St Louis, MO, USA) or saline was administered subcutaneously immediately after MCA occlusion^45^. Ifenprodil (10 mg/kg, Sigma-Aldrich) or saline was administered intraperitoneally immediately after MCA occlusion^36^.

### Functional outcome

Neurological deficits were assessed by an investigator blinded to the treatment of the animals 48 hours after MCA occlusion. Neurologic scores were assigned as follows: 0, no neurological deficit; 1, failure to fully extend left forepaw; 2, reduced resistance to lateral push; 3, spontaneous circling to left; 4, absence of spontaneous movement or unconsciousness. For evaluation of locomotor activity, the open field test were performed as described previously^16^,^46^.

### Measurements of infarct volume

At 48 hours after MCA occlusion, mice were anesthetized with chloral hydrate and sacrificed. The brains were removed and cut into eight coronal sections of 1-mm thickness using a mouse brain slice matrix. The sections were stained with 2% triphenyl-2,3,4-tetrazolium-chloride (TTC, Sigma-Aldrich) in PBS for 30 minutes. Sections were digitalized and infarct areas were measured blindly using the NIH Image J software.

### Western blot analysis

At 24 hours after MCA occlusion, mice were anesthetized, transcardially perfused with ice-cold PBS and their brains were removed. The tissues were lysed in RIPA buffer (Merck Millipore Billerica, MA, USA) with protease inhibitor cocktail (Roche Diagnostics, Indianapolis, IN, USA) and used for western blot as previously described^16^,^46^. The primary antibodies were rabbit anti-LC3 (Light Chain 3, 4108), rabbit anti-pERK1/2 (phosphorylation of extracellular signal-regulated kinase1/2, 9101), rabbit anti-pJNK (c-Jun N-terminal kinases, 9251), rabbit anti-p-p38 (9211), rabbit anti-NR1 (N-methyl-D-aspartate receptor, 4204), rabbit anti-NR2A (4205), rabbit anti-NR2B (4207), rabbit anti-β-actin (4967) (all from Cell Signaling Technology, Beverly, MA, USA), mouse anti-α-fodrin (AA6, Enzo Life Sciences, Inc. Farmingdale, NY, USA), rabbit anti-tPA (ASHTPA-GF-HT, Molecular Innovations Inc., Novi, MI, USA). In preliminary experiments, we could not achieve satisfactory western blot and immunoprecipitation in extracts from the ischemic or non-ischemic hemispheres of mice for ADAMTS13 with a couple of commercially available antibodies and thus coimmunoprecipitation of ADAMTS13 with NR2B and tPA were precluded from the study.

### Coimmunoprecipitation

Ischemic hemispheric brain tissues was homogenized in ice-cold RIPA lysis buffer (Merck Millipore) and proteinase inhibitor mixture (Roche Diagnostics). After clearing debris by centrifugation at 14,000 × g, protein concentration was determined using the BCA protein assay (Thermo Scientific, MA, USA). The lysate (500 μg) was incubated with anti-NR2B (2 μg; Millipore) or anti-tPA (2 μg; Molecular Innovations Inc.) antibody or normal IgG (2 μg, negative control) on a rotator at overnight 4 °C. Then, 20 μl Protein A agarose beads (Roche Diagnostics) were added and reacted for 6 hours at 4 °C. After washing with lysis buffer 3 times, the precipitates were used for Western blot.

### Immunohistochemistry

Mice were anesthetized deeply by chloral hydrate and perfused with PBS followed by 4% paraformaldehyde in PBS, pH 7.4. The brains were removed and postfixed in 4% paraformaldehyde, and then were immersed in 30% sucrose in PBS at 4 °C overnight. The tissues were frozen quickly and 20 μm sections were cut on a cryostat (Leica Microsystems Inc., Buffalo Grove, IL, USA). Immunohistochemistry was performed as described previously^39^. The primary antibodies were rabbit anti-fibrinogen (AP00766PU-N, Acris Antibodies, Inc. San Diego, CA, USA), rat anti-CD31 (PECAM-1, BD Pharmingen, San Diego, CA), and rabbit anti-LC3 (4108, Cell Signaling Technology). The secondary antibodies were Alexa Fluor 488 or 594 donkey anti-rabbit or anti-rat immunoglobulin G (IgG; Invitrogen, Camarillo, CA, USA). Nuclei were stained with DAPI. Images were obtained using an Olympus BX 51 microscope and an Olympus FV 1,000 confocal microscope.

### TUNEL staining

TUNEL (terminal deoxynucleotidyl transferase-mediated dUTP nick-end labeling) was performed on brain sections using *in situ* cell death detection kit (Roche Diagnostics) according to the manufacture’s instructions^46^.

### Fluoro-Jade B staining

Fluoro-Jade B (FJB) staining was performed as described previously^16^,^46^. In brief, cerebral tissue sections were kept in 0.06% potassium permanganate for 10 minutes and rinsed in distilled water. The sections were stained in 0.0004% Fluoro-Jade B solution (Merck Millipore) in 0.1% acetic acid, then rinsed in distilled water. After being dried, sections were cleared into xylene and coverslipped.

### Cell counting

For quantification of LC3, TUNEL, and FJB-labeled cells in the peri-infarct area, six fields from each section were captured under 40X objective. The total numbers of positive cells in the traced area were counted blindly with Image J software and expressed as per mm^2^. Three sections (2 mm apart) per mouse were analyzed from 5 mice of each group.

### Statistical analysis

Statistical analyses were performed using GraphPad Prism 6.0. Values are represented as mean ± SD. One-way analysis of variance (ANOVA, when the data were normally distributed) followed by the Boneferroni’s multiple comparison test was applied to western blot quantification of fibrin, fodrin, LC3-II/LC3-I, pERK1/2, anti-pJNK, p-p38, NR1, NR2A and NR2B, quantification of TUNEL, Fluoro-Jade B and LC3 staining, and quantification of coimmunoprecipitation experiments. Kruskal–Wallis test (when the data were not normally distributed) followed by the Dunn’s multiple comparison test was applied to infarct volume, neurologic scores and open field test. Mann-Whitney test was used to infarct volume when two groups were compared. P < 0.05 was considered statistically significant.

## Additional Information

**How to cite this article**: Fan, M. *et al.* Tissue Plasminogen Activator Neurotoxicity is Neutralized by Recombinant ADAMTS 13. *Sci. Rep.* **6**, 25971; doi: 10.1038/srep25971 (2016).
